# Structural Characterization of a Low Molecular Weight HG-Type Pectin From Gougunao Green Tea

**DOI:** 10.3389/fnut.2022.878249

**Published:** 2022-04-13

**Authors:** Tao Hong, Jiaying Zhao, Junyi Yin, Shaoping Nie, Mingyong Xie

**Affiliations:** State Key Laboratory of Food Science and Technology, China-Canada Joint Lab of Food Science and Technology, Key Laboratory of Bioactive Polysaccharides of Jiangxi Province, Nanchang University, Nanchang, China

**Keywords:** tea, homogalacturonan, rhamnogalacturonan I, structure, methylation, NMR

## Abstract

Tea is a popular beverage with a long history of safe and healthy use. Tea polysaccharide is a bioactive component extracted from tea, which has attracted more and more attention in recent decades. In this article, an acidic polysaccharide Gougunao tea polysaccharide (GPS) was isolated from Gougunao green tea by hot water extraction and ethanol precipitation. After purification by a diethylaminoethyl (DEAE) Sepharose Fast Flow column and a Sephacryl S-400 column, several homogalacturonan (HG) and rhamnogalacturonan-I (RG-I) fractions were obtained. Fraction GPS2b with the highest yield was selected for structural characterization by methylation and nuclear magnetic resonance (NMR) analysis. GPS2b was found to be an HG-type pectic polysaccharide (degree of methyl esterification [DE], 51.6%) with low molecular weight (*M*_*w*_, 36.8 kDa). It was mainly composed of →4)-α-Gal*p*A- (1→ and →4)-α-Gal*p*A-6-OMe-(1→. In addition, a minor highly branched RG-I domain was identified in this fraction. The investigation of structural features of tea polysaccharides can provide insights to understand their structure-bioactivity relationship.

## Introduction

Tea, made from the buds or leaves of the plant *Camellia sinensis* L., is a worldwide beloved beverage. Except for its good taste and flavor, the health benefits of tea should not be neglected. The bioactivities include anti-oxidation (1), anti-aging (2), anti-viral activity (3), anti-inflammation (4), and hypoglycemic activities (5). Gougunao green tea is a famous tea, which is planted and manufactured in Jiangxi, China. It has been awarded the gold prize of the Panama-Pacific International Exposition in 1915 due to its excellent quality. Gougunao green tea was found to have anti-oxidant and hypoglycemic activities *in vitro* (6).

Tea polysaccharide is a bioactive component in tea. The most important function of tea polysaccharide is hypoglycemic activity, which has been proved both *in vitro* (7, 8) and *in vivo* (5, 9). Additionally, tea polysaccharide has been found to exhibit numerous bioactivities, including anti-oxidation, anti-diabetic, anti-obesity, anti-tumor, and gut microbiota regulation effects (10). Structure analysis of tea polysaccharides is the foundation to understand its structure-bioactivity relationship (11). For example, tea polysaccharides with different structural features were found to display different hypoglycemic activities *in vitro* (9). Zhao et al. found that tea polysaccharides with different molecular weights (*M*w) displayed different protective effects on cells and different inhibition abilities of crystal adhesion (12).

However, the structure of tea polysaccharides reported pervious varied a lot as a result of different origins, varieties, and preparation methods. For example, two main glucose-containing polysaccharides were found in selenium-enriched tea from Ankang, Shaanxi (13). However, a complex arabinogalactan protein and a highly branched heteroxylan were characterized in both green and black teas from Curitiba, Brazil (14). In addition, pectic polysaccharides were also found in tea. Two homogalacturonan (HG) pectins were found to exist in Wufeng Green Tea from Hubei, China (15). Arabinogalactans were also characterized in Wufeng Green Tea and instant green tea from India (16, 17). Two distinct pectin fractions that contain rhamnogalacturonan (RG)-I and RG-II sequences were purified in green tea from Fujian, China (18).

In this article, tea polysaccharide was extracted by hot water from Gougunao green tea. A diethylaminoethyl (DEAE) Sepharose Fast Flow column and a Sephacryl S-400 column were applied for the purification of tea polysaccharides. The chemical composition and structural features of a main fraction, GPS2b, were elucidated. This research is aimed to provide structural information on tea polysaccharides and establish the foundation of their structure-bioactivity relationship.

## Materials and Methods

### Materials and Reagents

Gougunao green tea was purchased from a local market in Ji’an, Jiangxi province. D-galacturonic acid (GalA), D-arabinose (Ara), D-galactose (Gal), L-rhamnose (Rha), D-glucose (Glu), L-fucose (Fuc), D-fructose (Fru), sodium borodeuteride (NaBD_4_), deuterium oxide (D_2_O), carbodiimide, imidazole, and 4-morpholineethanesulfonic acid and methyl iodide (CH_3_I) were offered by Sigma-Aldrich Co. (St. Louis, MO, United States). D-Ribose (Rib), D-glucuronic acid (GlcA), dimethyl sulfoxide, xylene brilliant cyanine G, trifluoroacetic acid, and methylene chloride were obtained from Aladdin Biochemical Technology Co. (Shanghai, China). D-xylose (Xyl) and D-mannose (Man) were purchased from J&K Scientific Co. (Beijing, China). Sodium borohydride (NaBH_4_) was obtained from Kemiou Chemical Reagent Co. (Tianjin, China). DEAE Sepharose Fast Flow media were offered by Cytiva Co. (Marlborough, MA, United States). Acetic anhydride was provided by the Rich Joint Chemical Reagent Co. (Shanghai, China). All other reagents (ethanol, chloroform, N-butanol, sodium chloride, sulfuric acid, carbazole, sodium acetate, sodium hydroxide, hydrochloric acid, acetic acid, and ammonia solution) were of analytical grade and purchased from Sinopharm Chemical Reagent Co. (Shanghai, China).

### Extraction and Purification of Polysaccharides

**FIGURE 1:**
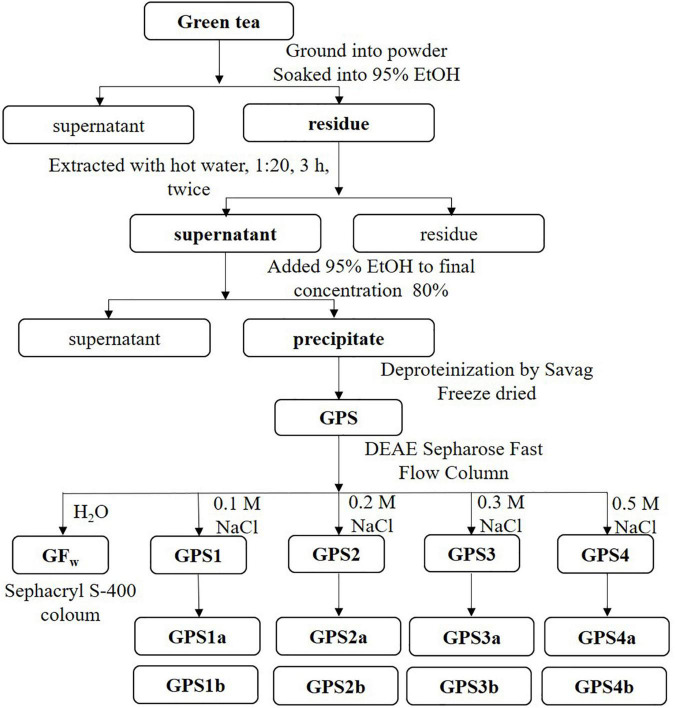
Flowchart of the extraction and purification process of polysaccharides from Gougunao green tea.

The extraction and purification process of tea polysaccharides are shown in Figure 1. Briefly, dried tea leaves were ground and sieved through a 60-mesh sieve, then immerged into 95% ethanol to remove alcohol-soluble ingredients, such as tea polyphenols. The residue was extracted with deionized water (95°C, 3 h) twice. After centrifugation, the supernatants were combined and concentrated to half the original volume under reduced pressure at 60°C. Ethanol was added for the precipitation of polysaccharides. The precipitate was treated with a Sevag reagent to remove the protein (19). Finally, the liquid was dialyzed in dialysis bags (8,000–14,000 Da cutoff), concentrated, and lyophilized to get GPS.

Gougunao tea polysaccharide (1 g) was dissolved in 20 ml of distilled water and centrifuged (15,000 *g*, 10 min). The supernatant was applied to a DEAE Sepharose Fast Flow column (Ø45 × 600 mm). The GPS was eluted with distilled water at first, and then with NaCl at different concentrations, successively. Different fractions were collected, concentrated, dialyzed, and freeze-dried, which were labeled as GFw, GPS1, GPS2, GPS3, and GPS4. After that, GPS1, GPS2, GPS3, and GPS4 were redissolved in 0.1 M NaCl and further purified on a High-Resolution Sephacryl™ S-400 column (Ø26 × 600 mm, Cytiva, Marlborough, MA United States). This purification process was monitored by the refractive index detector (RID) (RI-102, SHOKO, Japan).

### Structural Analysis of Polysaccharides

#### Determination of Uronic Acid Content

Acidic sugar content was measured by the carbazole-sulfuric acid method with GalA as the standard (20).

#### Homogeneity Analysis and Molecular Weight Determination

Homogeneity of different fractions and the average molecular weight (*M*_*w*_) were evaluated by the high-performance gel permeation chromatography (HPGPC) (Waters, Milford, MA, United States). The system was equipped with an Ultrahydrogel™ Guard Column (Ø6 × 40 mm) and an Ultrahydrogel™ Linear Column (Ø7.8 mm × 300 mm) (Waters, Milford, MA, United States). The mobile phase was 0.1 M NaCl, and the elution rate was 0.6 ml/min (21).

#### Monosaccharide Composition Analysis

Polysaccharides were firstly hydrolyzed with 12 M H_2_SO_4_ for 0.5 h and then with 2 M H_2_SO_4_ at 100°C for 3 h (22). The **hydrolyzate** was diluted to be 20 μg/ml and analyzed by a high-performance anion-exchange chromatography with pulsed amperometric detection (Dionex ICS-5000, Thermo Fisher, Waltham, MA, United States). A CarboPac™ PA20 Guard Column (Ø3 mm × 30 mm, Thermo Fisher, Waltham, MA, United States) and CarboPac™ PA20 Analytical Column (Ø3 mm × 150 mm, Thermo Fisher, Waltham, MA, United States) were equipped in series and eluted with the mobile phase at 0.5 ml/min (23).

#### Fourier Transform Infrared Spectroscopy

All IR spectra were obtained by a Nicolet IS50 fourier transform infrared (FT-IR) spectrophotometer (Thermo Fisher, Waltham, MA, United States) under the attenuated total reflection (ATR) mode at 4 cm^–1^ resolution by accumulating 64 scans. The scanned wave number range was between 4,000 and 400 cm^–1^.

#### Carboxyl Reduction and Methylation Analysis

Because of the high content of GalA in the sample, the uronic acid was reduced before methylation analysis. To distinguish Gal, GalA, and esterified GalA, a carboxyl reduction method of Kim and Carpita (24) was adopted. At first, the sample was dissolved into imidazole HCl. NaBD_4_ was added for the reduction of esterified GalA. After that, acetic acid was added to destroy excess reductant. The sample was dialyzed and freeze-dried. Esterified GalA was reduced to Gal and labeled by deuterium during this step. At the second step, carbodiimide was added to activated GalA. The sample was split equally into two tubes. NaBH_4_ and NaBD_4_ were added into different tubes for the labeling of GalA, respectively. The D/H-labeled sample and the D/D-labeled sample were dialyzed, lyophilized, and finally subjected to methylation analysis.

The linkage pattern of GPS2b was analyzed by a slightly modified method based on Pettolino et al. (25). Firstly, methanol was added to the uronic acid reduced sample, and the mixture was dried with a stream of nitrogen for dehydration. The dried sample was dissolved in anhydrous dimethyl sulfoxide (DMSO). A slurry of NaOH in DMSO was added to the solution to create an alkaline environment. CH_3_I was added and then sonicated to enable the complete methylation of polysaccharides. Distilled water was added to stop the methylation reaction and the methylated sample was extracted with CH_2_Cl_2_. After hydrolysis and acetylation, the partially methylated alditol acetates (PMAAs) were obtained. The PMAAs were analyzed by an Agilent 7890B/7000D GC-MS system (Agilent, Santa Clara, CA, United States) equipped with an SP-2330 column (Sigma-Aldrich, St. Louis, MO, United States) (23).

#### Nuclear Magnetic Resonance Spectroscopy

Fraction GPS2b was dissolved in D_2_O, stirred overnight, and lyophilized. This treatment was repeated three times for the complete deuterium exchange. After that, the sample was dissolved in D_2_O for the 1D and 2D NMR tests by a Bruker AVANCE III HD 400 MHz NMR spectrometer (Bruker, Zurich, Switzerland). 1D (^1^H and ^13^C) NMR spectra and 2D NMR spectra, including ^1^H-^13^C heteronuclear single-quantum coherence spectroscopy (HSQC), ^1^H-^13^C heteronuclear multiple-bond spectroscopy (HMBC), ^1^H-^1^H correlation spectroscopy (COSY), ^1^H-^1^H total correlation spectroscopy (TOCSY), and ^1^H-^1^H nuclear overhauser effect spectroscopy (NOESY), were recorded at 298 K.

#### Statistical Analysis

One-way analysis of variance (ANOVA) with Turkey’s multiple range test using SPSS 26.0 (IBM, Armonk, NY, United States) was applied for the processing of data expressed as mean ± SD (*n* = 3).

## Results

### Extraction, Characterization, and Purification of Gougunao Tea Polysaccharide

**FIGURE 2:**
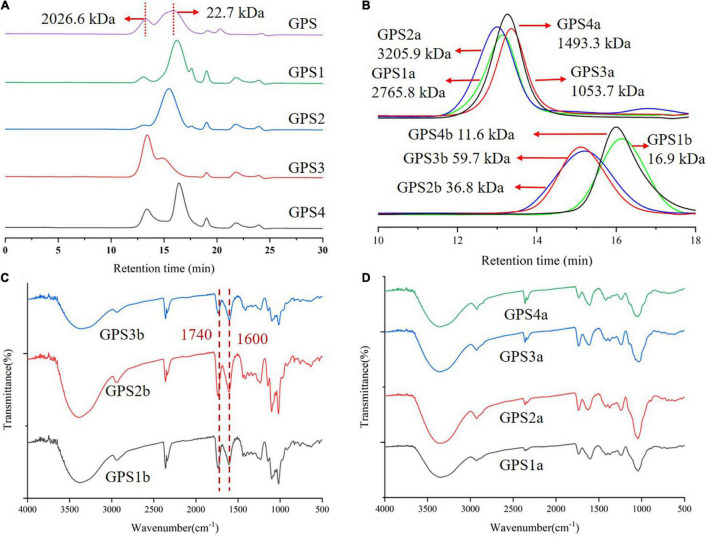
**(A)** High-performance gel permeation chromatography (HPGPC) elution profile of Gougunao tea polysaccharide (GPS) and its DEAE Sepharose column purified fractions; **(B)** HPGPC elution profile of Sephacryl S-400 column purified fractions; **(C)** Infrared (IR) spectrum of GPS1b, GPS2b, and GPS3b. **(D)** IR spectrum of GPS1a, GPS2a, GPS3a, and GPS4b.

Gougunao tea polysaccharide was extracted from Gougunao green tea by hot water. The yield of GPS was found to be 3.18%. As shown in Figure 2A, the HPGPC chromatogram suggests that GPS exhibits polydispersity characteristics. There were two main peaks in the HPGPC profile of GPS, corresponding to the *M*w of 2026.6 and 22.7 kDa, respectively. Monosaccharide analysis result indicated that GPS was mainly constituted by GalA, Gal, and Ara, which was consistent with previous reports (16). The IR spectrum of GPS (Supplementary Figure 1) showed characteristic absorptions of pectic polysaccharides, with bands at 1,740 and 1,600 cm^–1^ indicating characteristic absorption peaks of partial methyl-esterified pectin, which was confirmed by the ^13^C NMR spectrum (signals at 173.39 and 170.72 ppm) as shown in Supplementary Figure 2.

Due to the polydispersity and high uronic acid content, GPS was firstly purified by a DEAE anion-exchange column using NaCl solutions at different concentrations. As shown in Figure 2A, the HPGPC chromatograms of DEAE-purified fractions (GPS1, GPS2, GPS3, and GPS4) are not homogeneous. Thus, further purification of these fractions was performed by a gel permeation column. HPGPC analysis and monosaccharide composition analysis were used to monitor this purification process. A total of eight homogeneous fractions were obtained (Figure 2B). Their uronic acid contents were analyzed and are shown in Supplementary Table 1. It was found that fractions GPS1b, GPS2b, and GPS3b were mainly composed of uronic acid, while the uronic acid contents of GPS1a, GPS2a, GPS3a, and GPS4a were relatively low.

### Monosaccharide Compositions Analysis

The monosaccharide composition results of GPS and eight purified fractions are summarized in Table 1. These results indicated that these purified fractions except for GPS4b were mainly composed of GalA, Gal, and Ara, displaying distinct features of pectic polysaccharides. Pectic polysaccharide is constituted by different structural domains, mainly including HG and RG-I domains. The molar ratio of these domains in different fractions could be estimated by their monosaccharide composition (26). According to the monosaccharide composition, GPS1a, GPS2a, GPS3a, and GPS4a were mainly composed of RG-I domain, while HG domain was the main constituent among GPS1b, GPS2b, and GPS3b. The relatively low content of Rha and higher amount of Ara and Gal indicated RG-I domain in these fractions might be highly branched. Combining the aforementioned results, GPS2b with the highest yield was chosen for detailed structural analysis.

**TABLE 1:** Monosaccharide compositions of Gougunao tea polysaccharide (GPS) and its purified fractions (expressed as mol%).

Monosaccharides	GPS	GPS1a	GPS2a	GPS3a	GPS4a	GPS1b	GPS2b	GPS3b	GPS4b
Rha	5.1	13.0	8.3	15.7	13.8	0.1	2.4	2.3	5.5
Ara	18.4	32.0	28.0	32.6	30.4	12.2	6.0	9.2	ND^c^
Gal	21.6	38.9	49.8	24.2	38.5	8.1	3.7	8.6	1.4
Glc	1.2	0.1	2.1	ND	0.2	ND	ND	ND	ND
Rib	6.7	ND	ND	ND	ND	ND	ND	ND	85.1
GalA	47.0	15.6	11.7	27.2	16.5	79.6	87.9	79.9	7.9
HG*^a^*	41.8	2.7	3.4	11.4	2.7	79.5	85.5	77.5	ND
RG-I*^b^*	50.3	96.9	94.3	88.3	96.5	20.5	14.5	22.5	ND

*^a^HG = GalA-Rha.*

*^b^RG-I = [GalA-HG] + Rha + Ara + Gal.*

*^c^ND: not detected.*

### Fourier Transform Infrared Analysis of GPS2b

Fourier transform infrared spectrum of GPS2b displayed typical features of HG pectin (Figure 2C). The strong absorption at about 3,370 cm^–1^ could be assigned to -OH stretching in polysaccharides. A moderate band at around 2,940 cm^–1^ was attributed to the stretching of -CH groups (27). The COO- doublet, which was characteristic for pectin at 1,740 and 1,600 cm^–1^, could be clearly observed, corresponding to C = O vibration of methyl-esterified carboxyl groups (COO-R) and ionic carboxyl groups (COO-), respectively (28). Three absorption peaks in the “fingerprint” regions at 1,144, 1,100, and 1,017 cm^–1^ indicated that sugars in GPS2b existed as pyranose forms. Additionally, absorptions at around 832 and 916 cm^–1^ indicated that the present form of anomeric carbon was mainly α configuration (29). FT-IR spectra of other fractions were displayed in Figures 2C,D.

### Linkage Pattern of Fraction GPS2b

**FIGURE 3:**
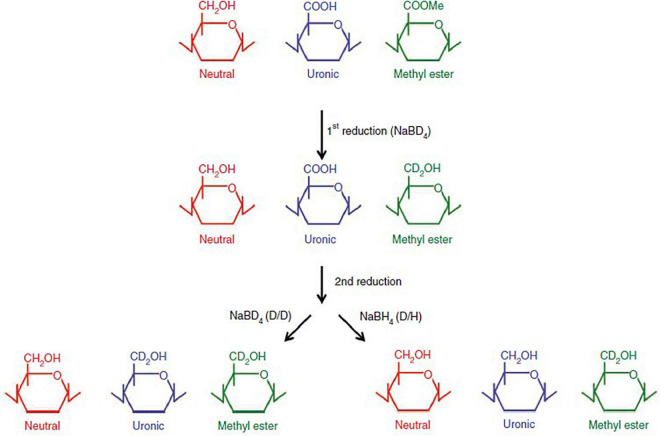
Deuterium labeling of galacturonic acid (GalA) and methyl-esterified GalA during a two-step reduction procedure, reprinted with the permission from Springer Nature (25).

Fraction GPS2b was subjected to methylation analysis for the linkage patterns analysis. It was reduced prior to methylation because of the existence of high content of uronic acids (30). Deuterium is used for labeling during the reduction procedure for the differentiation of Gal, GalA, and methyl-esterified GalA, as shown in Figure 3.

As shown in Table 2, GPS2b is mainly composed of 1,4-linked Gal*p*A and methyl-esterified 1,4-linked Gal*p*A, with a ratio of about 1:1. A small amount of T-Gal*p*A and methyl-esterified T-linked Gal*p*A were identified. In addition, trace amounts of T-Rha*p*, 1,2-linked Rha*p*, and 1,2,4-linked Rha*p* in RG-I backbone were found in methylation analysis, which was consistent with the result of monosaccharide analysis. The existence of 1,5-linked Ara*f* and 1,4-Gal*p* was considered to be originated from the AG-I side chain of RG-I. Residues 1,3,6-Gal*p*, 1,6-Gal*p*, and T-Ara*f* could be attributed to the AG-II side chain of RG-I. The amount of these residues was relatively low, which was in accordance with the monosaccharide analysis result and confirmed the relatively low abundance of RG-I domain in GPS2b. The mass spectra of residues 1,3,4-Gal*p* (2.3%), 1,2,4-Gal*p* (1.2%), and 1,4,6-Gal*p* (1.8%) showed that they were deuterium-labeled, indicating they originated from GalA or methyl-esterified GalA. This was unreasonable because GalA in HG pectin was generally not branched and GalA cannot branch at C6. A possible explanation for this phenomenon was that these residues originated from the incompletely methylated GalA.

**TABLE 2:** Linkage analysis of GPS2b.

Monosaccharide	PMAA	Linkage	Molar ratio (%)
Rha	2,3,4-Me_3_ Rha	T-Rha*p*	tr.
	3,4-Me_2_ Rha	1,2-Rha*p*	tr.
	3-Me Rha	1,2,4-Rha*p*	tr.
Ara	2,3,5-Me_3_ Ara	T-Ara*f*	2.55
	2,3-Me_2_ Ara	1,5-Ara*f*	tr.
Gal	2,3,4,6-Me_4_ Gal	T-Gal*p*	0.9
	2,3,6-Me_3_ Gal	1,4-Gal*p*	1.4
	2,3,4-Me_3_ Gal	1,6-Gal*p*	tr.
	2,4-Me_2_ Gal	1,3,6-Gal*p*	2.2
GalA	2,3,4,6-Me_4_ Gal	T-Gal*p*A	3.2
	2,3,4,6-Me_4_ Gal	T-MeGal*p*A	2.9
	2,3,6-Me_3_ Gal	1,4-Gal*p*A	43.8
	2,3,6-Me_3_ Gal	1,4-MeGal*p*A	37.7

*tr.: Lower than limitation of quantification.*

### Nuclear Magnetic Resonance of Fraction GPS2b

**FIGURE 4:**
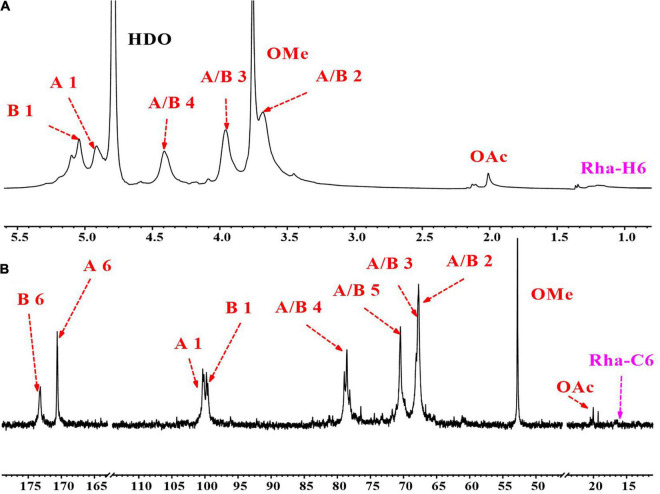
1D nuclear magnetic resonance (NMR) spectra of GPS2b. **(A)** ^1^H NMR spectrum; **(B)** ^13^C NMR spectrum.

**FIGURE 5:**
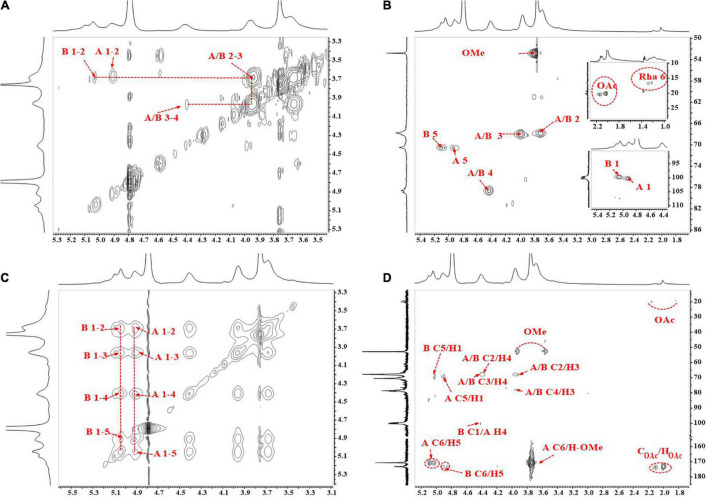
2D nuclear magnetic resonance (NMR) spectra of GPS2b. **(A)** ^1^H-^1^H correlation spectroscopy (COSY) spectrum; **(B)** HSQC spectrum; **(C)** ^1^H-^1^H nuclear overhauser effect spectroscopy (NOESY) spectrum; **(D)**^ 1^H-^13^C heteronuclear multiple-bond spectroscopy (HMBC) spectrum.

The 1D (^1^H and ^13^C) and 2D (COSY, HSQC, HMBC, and NOESY) NMR spectra of GPS2b are shown in Figures 4, 5, respectively. TOCSY spectrum of GPS2b is shown in Supplementary Figure 3. Two anomeric signals at 5.05 and 4.91 ppm in the ^1^H spectrum indicated the α configuration of anomeric protons. These signals were assigned to be anomeric protons of →4)-α-Gal*p*A-6-OMe-(1 → and →4)-α-Gal*p*A-(1 → residues, respectively. Signals of non-reducing terminal α-D-Gal*p*A-(1→… residue was also observed. In addition, weak signals of C1/H1 cross-peaks of Ara and Gal were found in the anomeric region of the HSQC spectrum.

#### Homogalacturonan Backbone

##### Residue →4)-α-Gal*p*A-6-OMe-(1→ and →4)-α-Gal*p*A- (1→

There were two high-intensity signals at the anomeric region of the HSQC spectrum. Combining results of monosaccharide analysis and methylation analysis, cross-peaks at 99.75/5.05 and 100.27/4.91 ppm could be assigned to C1/H1 of →4)-α-Gal*p*A-(1→ (**GA**_4_) and →4)-α-Gal*p*A-6-OMe-(1→ (**mGA**_4_) residues, respectively. The correlations from H2 to H4 can be readily observed in the COSY spectrum. These signals of **mGA**_4_ were identical with those of **GA**_4_. Carboxylic and methyl-esterified carboxylic C6 signals of **GA**_4_ and **mGA**_4_ were reported to be located at the downfield region in the ^13^C spectrum. Signals at 173.24 and 170.68 ppm were assigned to C6 of **GA**_4_ and **mGA**_4_. The H5s of **GA**_4_ at 4.91 and 4.85 ppm and H5s of **mGA**_4_ at 5.03 and 5.09 ppm were readily obtained from the HMBC spectrum according to the C6/H5 coupling. Since H2 to H5 were revealed, the corresponding C2 to C5 of **GA**_4_ and **mGA**_4_ were found from the HSQC spectrum. The chemical shift of these protons and carbons, which is shown in Table 3, is consistent with previous studies (31, 32).

**TABLE 3:** The chemical shift of residues in homogalacturonan (HG) backbone.

Residues		C1/H1	C2/H2	C3/H3	C4/H4	C5/H5	C6/H6	OMe
→4)-α-GalpA-6-OMe-(1→	A	100.27	67.77	67.88	78.60	70.42	170.68	52.78
		4.91	3.69	3.97	4.42	5.09/5.03	/	3.76
→4)-α-GalpA-(1→	B	99.75	67.77	67.88	78.60	70.46	173.24	
		5.05	3.69	3.97	4.42	4.91/4.85	/	

The correlation of methyl ester carbon and proton from **mGA**_4_ at 52.78/3.76 ppm was clearly observed in the HSQC spectrum. Two symmetrical cross-peaks at 52.78/3.93 and 52.78/3.56 ppm in the HMBC spectrum confirmed the correlation of methyl ester carbon at 52.78 ppm and methyl ester protons at 3.76 ppm. The chemical shift of C6 of **mGA**_4_ was validated by the cross-peak of C6/H_*OMe*_ at 170.68/3.76 ppm in the HMBC spectrum. The intraresidual coupling of both residues can also be confirmed by cross-peaks of C2/H3 at 67.77/3.97 ppm, C2/H4 at 67.77/4.42 ppm, C3/H4 at 67.88/4.42 ppm, and C4/H3 at 78.60/3.97 ppm in the HMBC spectrum. Additionally, cross-peaks of H1/(H2–H5) in TOCSY and NOESY also validated this coupling. Inter-residual coupling of **GA**_4_ and **mGA**_4_ was confirmed by a cross-peak of **mGA**_4_ C1/**GA**_4_ H4 at 100.27/4.42 ppm in HMBC spectrum and **GA**_4_ H4/**mGA**_4_ H1 and **mGA**_4_ H4/**GA**_4_ H1 at 4.42/4.91 and 4.42/5.03 ppm in NOESY spectrum.

##### Residue α-Gal*p*A-(1→

Anomeric signal of terminal residue α **-Gal*p*A-(1→ (GAt)** was overlapped with the anomeric signal of **GA**_4_, so do C2/H2 and C3/H3. But **GA**_*t*_ could be distinguished from C4/H4 and C5/H5 signals at 70.14/4.27 and 70.71/4.71 ppm in the HSQC spectrum, which was consistent with previous reports (33).

##### Residue Acetylated GalpA

The signals of methyl carbon from acetyl groups were identified at 20.39 and 20.04 ppm in the ^13^C NMR spectrum and signals of protons from acetyl groups were found at 2.13 and 2.02 ppm in the ^1^H NMR spectrum. Their correlation was observed in the HSQC spectrum. In addition, the correlation of these protons and carbonyl carbon from the acetyl group at 173.75 ppm was also observed in the HMBC spectrum. Besides, the NOESY spectrum displayed the correlation between protons from the acetyl groups and protons from Gal*p*A residues. These all indicated the existence of acetyl-substituted Gal*p*A in GPS2b.

#### Rhamnogalacturonan-I Side Chains

^13^C signals located at the downfield area (from 105 to 110 ppm) in the anomeric region of the ^13^C spectrum and HSQC spectrum could be attributed to anomeric carbon signals of Ara residues. Three cross-peaks at 107.36/5.05 and 109.20/5.20 ppm were found in this area, which could be attributed to anomeric signals of **→5)**-α -**Ara*f*-(1→ (A**_5_**) and terminal** α **-Ara*f*-(1→ (A**_*t*_**)**. Two anomeric signals at 104.30/4.60 and 102.94/4.50 ppm in the HSQC spectrum could be assigned to residues β **-Gal*p*-(1→ (G**_*t*_**)** and **→4)-**β **-Gal*p*-(1→ (G**_4_**)**. ^1^H signals at 1.22 and 1.28 ppm and ^13^C signals at 16.48 and 16.75 ppm indicated the existence of Rha in GPS2b. Their correlations were found in the HSQC spectrum. According to previous reports (33), cross-peaks of 16.48/1.22 and 16.75/1.28 ppm could be assigned to C6/H6 of **→2)-α - Rha*p*-(1→ (R_2_)** and **→2,4)-α - Rha*p*-(1→ (R_24_)** residues. Signals of other protons and carbons were not found because of the relatively low content of Rha in GPS2b, which was also be revealed by monosaccharide analysis and methylation analysis. The low content of the Rha residues and relatively higher content of Ara and Gal residues indicated the presence of highly branched side chains and the short length of the backbone region of RG-I, which confirmed the result of monosaccharide analysis.

Unfortunately, limited by the sensitivity and resolution of the NMR instrument and the relatively low proportion of these residues in GPS2b, some signals of them were masked by strong-intensity signals or failed to be reflected in NMR spectra. Thus, the intraresidual and inter-residual coupling of these residues was not clearly identified.

In conclusion, combining the results of methylation analysis and NMR analysis, GPS2b was a typical pectic polysaccharide mainly comprised of HG domain, which was constituted by**→ 4)-**α **-Gal*p*A-6-OMe-(1→** and **→ 4)-**α **-Gal*p*A- (1→** with a molar ratio of 1:1. In addition, a minor highly branched RG-I domain was identified in GPS2b.

## Discussion

*Camellia sinensis* L. is a dicotyledonous plant. The pectic polysaccharide was reported to be a major component in the primary cell wall of dicotyledon that plays an important role in plant growth and development. The result that GPS extracted from tea leaves was identified as pectic polysaccharide is consistent with other previous reports (14). The structural heterogeneity of pectic polysaccharides in plant cell wall has been well recognized (34). According to the “relative order structure” theory of polysaccharides, most polysaccharide structures can be classified into a particular type of chemical structure (35). Under the guidance of this theory, several HG pectin and RG-I pectin fractions were purified from GPS and preliminarily characterized by monosaccharide composition analysis.

Fraction GPS2b with the highest yield was selected for further characterization by methylation analysis and 1D/2D NMR. According to the results of deuterium-labeled methylation analysis and NMR spectra, GPS2b was confirmed as HG pectin with partially methyl esterification at the C-6 carboxyl groups. HG accounted for approximately 65% of pectin in the plant cell walls. Though the specific mechanism of pectin biosynthesis is not very clear, it is well recognized that pectin is synthesized in the Golgi apparatus. During the biosynthesis process, up to 80% of GalA in nascent HG can be methylated by methyltransferases. After secretion to the cell wall, the methyl ester groups can be removed from methylated HG by pectin methylesterases during maturing (36). Tea leaves are tender buds of the plant *Camellia sinesis* L. Thus, the methyl ester groups in green tea leaves are partially removed. In addition, a lesser extent of acetylation was found in GPS2b. It was reported that GalA units in pectin polysaccharides can be substituted by acetyl groups at O-2 and/or O-3. However, subject to a relatively low abundance of acetylated GalA residue, signals of other carbons and protons in this residue could not be identified. Thus, it was also unable to determine the site of O-acetate substitution. A minor highly branched RG-I domain was also found in GPS2b. Some researchers reported that different pectin domains were covalently linked to one another in the cell wall, forming an interconnected pectin net (37). However, due to the relatively low abundance of the RG-I domain, the evidence of the covalent connection of the RG-I domain and HG domain in this fraction was not identified. Future studies are required to confirm whether these domains are covalently linked. If so, are they arranged in a specific order or not?

Bioactivities and functional properties of HG pectin are closely related to their structural features, such as *M*w, HG amount, degree of polymerization (DP), and degree of methyle-sterification (DE). HPGPC results indicated that the *M*w of GPS2b was 36.8 kDa. The *M*w of GPS2b is higher than *M*w of two HGs extracted from Wufeng Green Tea (20 and 20 kDa) (15). However, it is much lower than commercial citrus pectins (164–235 kDa) (28). It was reported that HG with lower *M*w exhibited better fermentation properties, with high production of short-chain fatty acids and abundant SCFA-producer genera (38). Thus, it is worthwhile to explore the fermentation properties of this fraction in future studies. In addition, it was also reported that besides DE and *M*w, both HG amount and DP can also significantly affect the gelling properties of pectin (39). Thus, gelling properties of tea polysaccharides would be another interesting point for further exploration.

## Conclusion

**FIGURE 6:**
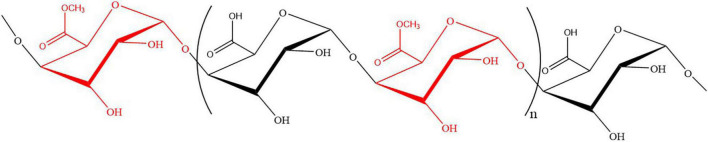
Homogalacturonan (HG) backbone of GPS2b.

An acidic polysaccharide was extracted by hot water from Gougunao green tea. Primary structural analysis showed that it was a mixture of pectic polysaccharides. After purification, several homogenous HG pectic polysaccharides and RG-I pectic polysaccharides were obtained. Due to the highest yield, fraction GPS2b was chosen for further analysis. Results of methylation analysis and NMR analysis confirmed the HG structure features of this fraction. It was mainly composed of **→4**)-α -**Gal*p*A-6-OMe-(1→** and **→4)**-α -**Gal*p*A- (1→**with a molar ratio of 1:1 (Figure 6). This study provided detailed structural features of HG pectin in tea leaves. However, because of the limitation of the relatively low yield, the structure of other fractions from GPS has not been elucidated. This research indicated that tea leaves could be considered as an alternative resource of pectin, which might have great potential in the food and health industry. Therefore, the bioactivities and industrial applications of tea polysaccharides would be worthwhile to be investigated.

## Data Availability Statement

The original contributions presented in the study are included in the article/Supplementary Material, further inquiries can be directed to the corresponding author.

## Author Contributions

TH: investigation and writing – review and editing. JZ: investigation. JY: supervision, funding acquisition, and writing – review and editing. SN and MX: resources, supervision, and funding acquisition. All authors contributed to the article and approved the submitted version.

## Conflict of Interest

The authors declare that the research was conducted in the absence of any commercial or financial relationships that could be construed as a potential conflict of interest.

## Publisher’s Note

All claims expressed in this article are solely those of the authors and do not necessarily represent those of their affiliated organizations, or those of the publisher, the editors and the reviewers. Any product that may be evaluated in this article, or claim that may be made by its manufacturer, is not guaranteed or endorsed by the publisher.
