# Supplementary material for: Structural Characterization of a Low Molecular Weight HG-Type Pectin From Gougunao Green Tea
Source: Front Nutr. 2022 Apr 13;9:878249. doi: 10.3389/fnut.2022.878249 (PMC9044067; doi:10.3389/fnut.2022.878249)
Supplement: Supplementary file 1 [file Data_Sheet_1.pdf]

Supplementary materials

Table S1. Yield and uronic acid content of GPS and purified fractions [#].

	Yield (%)	Uronic acid (%)
GPS	3.18 *	45.9±1.5[a]
GPS1	7.0 **	55.1±1.2[b]
GPS1a	5.6***	22.3±0.5[ef]
GPS1b	39.3 ***	77.6±1.2[g]
GPS2	38.1**	70.2±0.8[c]
GPS2a	2.3***	21.0±0.4[e]
GPS2b	38.4***	81.2±1.3[g]
GPS3	15.2 **	47.9±0.7[a]
GPS3a	19.5***	38.0±0.7[h]
GPS3b	17.9***	61.1±2.0[i]
GPS4	15.4 **	14.0±0.8[d]
GPS4a	9.3 ***	24.8±1.9[f]
GPS4b	36.9 ***	7.3±0.5[j]

[#] Data are expressed as mean ± SD (n = 3) except the yields.

*: calculated based on the dry weight of tea leaves.

**: calculated based on the weight of GPS.

***: calculated based on the weight of DEAE purified fractions.

[a-j] Values followed by the different letter in the same column are significantly different ($p < 0.05$).

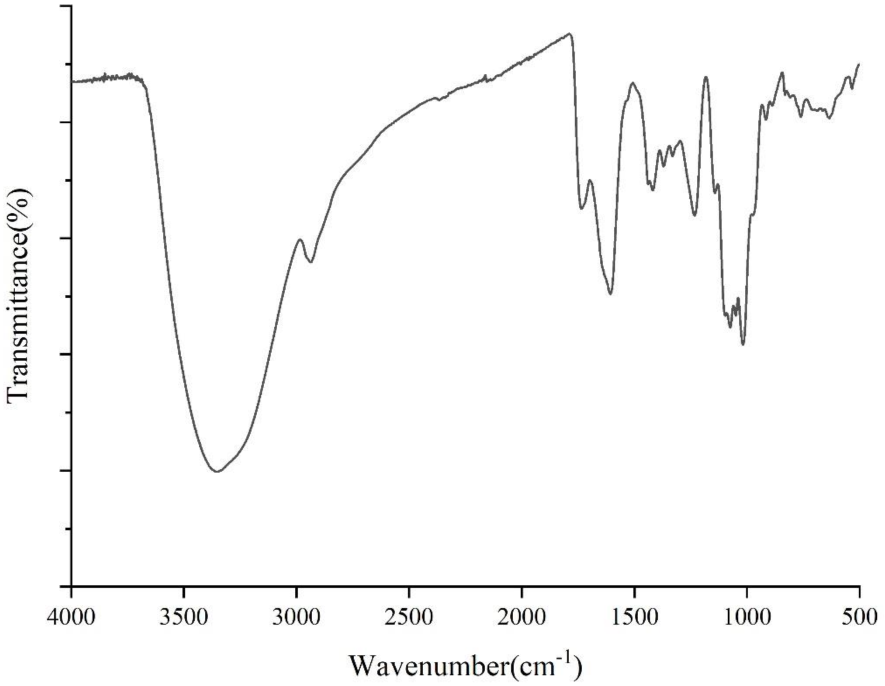

Figure S1. IR spectrum of GPS.

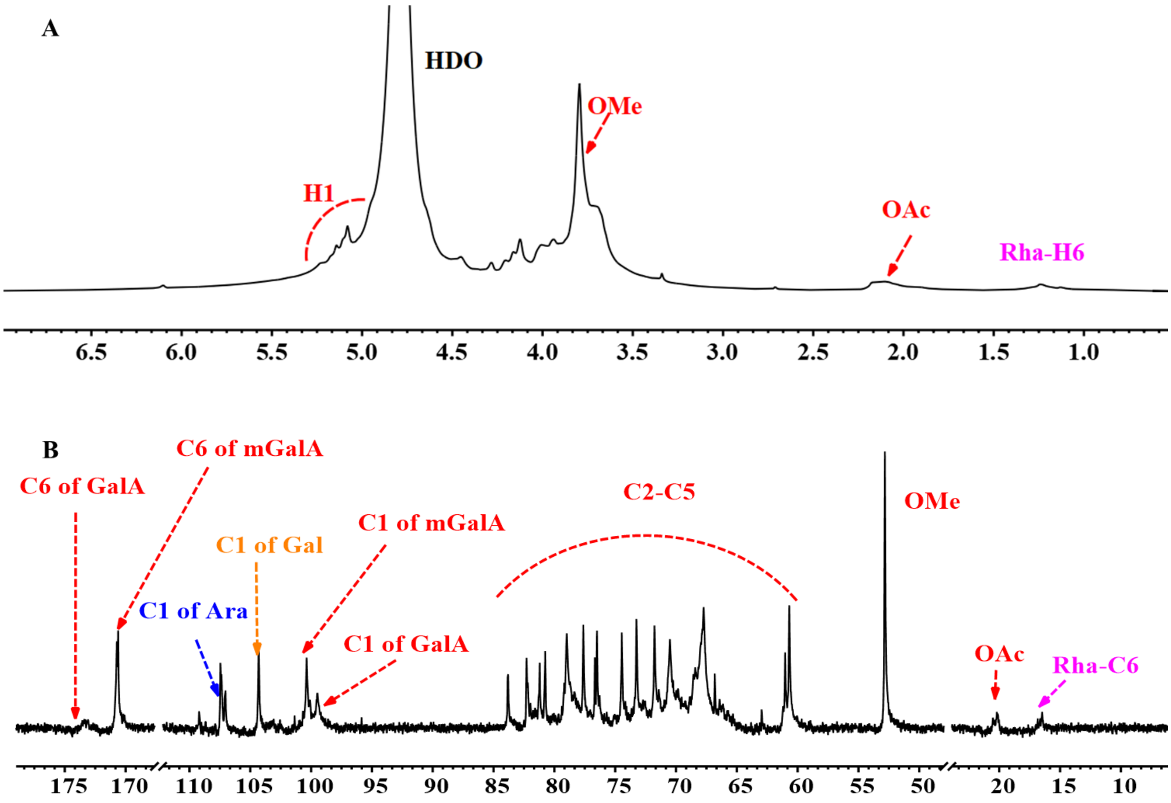

Figure S2. 1D NMR spectra of GPS. A. ^{1}H NMR spectrum; B. ^{13}C NMR spectrum.

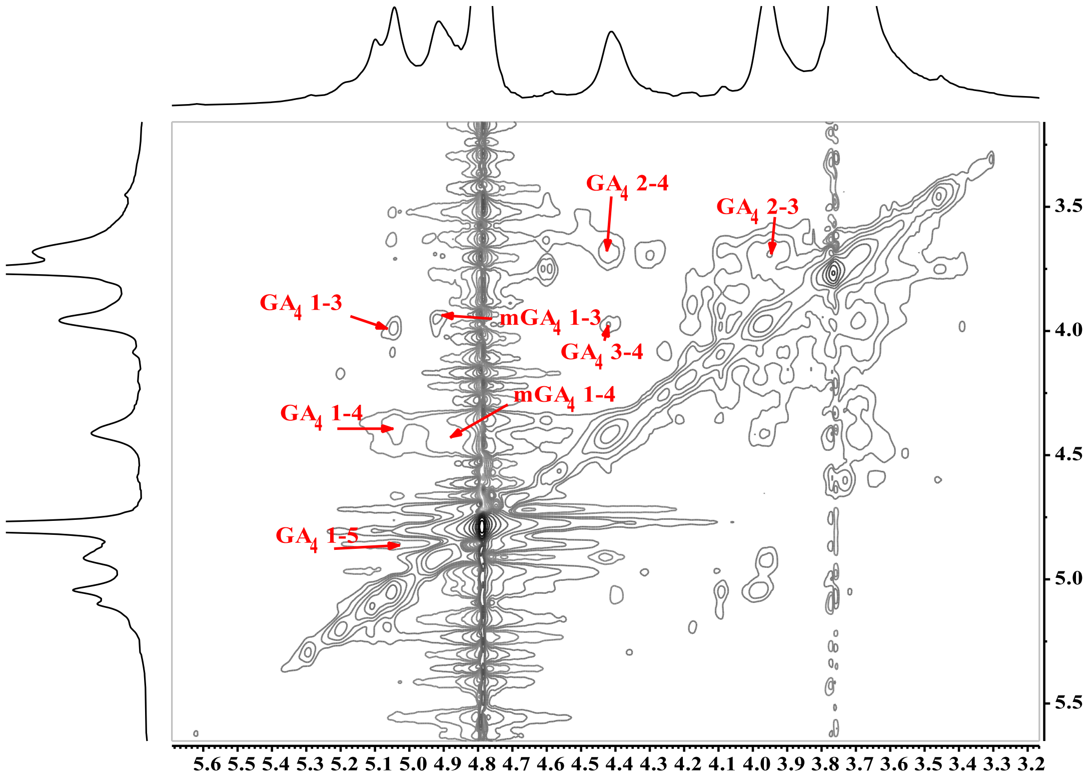

Figure S3. TOSCY spectrum of GPS2b.
